# Disclosure Patterns of Opioid Use Disorders in Perinatal Care During the Opioid Epidemic on X From 2019 to 2021: Thematic Analysis

**DOI:** 10.2196/52735

**Published:** 2024-10-07

**Authors:** Dezhi Wu, Minnie Ng, Saborny Sen Gupta, Phyllis Raynor, Youyou Tao, Yang Ren, Peiyin Hung, Shan Qiao, Jiajia Zhang, Jennifer Fillo, Xiaoming Li, Constance Guille, Kacey Eichelberger, Bankole Olatosi

**Affiliations:** 1 Department of Integrated Information Technology University of South Carolina Columbia, SC United States; 2 Department of Biobehavioral Health & Nursing Science College of Nursing University of South Carolina Columbia, SC United States; 3 Department of Information Systems and Business Analytics Loyola Marymount University Los Angeles, CA United States; 4 Department of Computer Science and Engineering University of South Carolina Columbia, SC United States; 5 Department of Health Services Policy and Management Arnold School of Public Health University of South Carolina Columbia, SC United States; 6 Department of Health Promotion, Education, and Behavior Arnold School of Public Health University of South Carolina Columbia, SC United States; 7 Department of Epidemiology and Biostatistics Arnold School of Public Health University of South Carolina Columbia, SC United States; 8 Department of Psychiatry and Behavioral Sciences College of Medicine Medical University of South Carolina Charleston, SC United States; 9 Department of Obstetrics and Gynecology University of South Carolina School of Medicine Greenville Prisma Health Greenville, SC United States

**Keywords:** X, Twitter, opioid use disorder, thematic analysis, pregnancy, perinatal care, women and child health, maternal health, COVID-19, opioid epidemic

## Abstract

**Background:**

In 2021, the United States experienced a 14% rise in fatal drug overdoses totaling 106,699 deaths, driven by harmful opioid use, particularly among individuals in the perinatal period who face increased risks associated with opioid use disorders (OUDs). Increased concerns about the impacts of escalating harmful opioid use among pregnant and postpartum persons are rising. Most of the current limited perinatal OUD studies were conducted using traditional methods, such as interviews and randomized controlled trials to understand OUD treatment, risk factors, and associated adverse effects. However, little is known about how social media data, such as X, formerly known as Twitter, can be leveraged to explore and identify broad perinatal OUD trends, disclosure and communication patterns, and public health surveillance about OUD in the perinatal period.

**Objective:**

The objective is 3-fold: first, we aim to identify key themes and trends in perinatal OUD discussions on platform X. Second, we explore user engagement patterns, including replying and retweeting behaviors. Third, we investigate computational methods that could potentially streamline and scale the labor-intensive manual annotation effort.

**Methods:**

We extracted 6 million raw perinatal-themed tweets posted by global X users during the opioid epidemic from May 2019 to October 2021. After data cleaning and sampling, we used 500 tweets related to OUD in the perinatal period by US X users for a thematic analysis using NVivo (Lumivero) software.

**Results:**

Seven major themes emerged from our thematic analysis: (1) political views related to harmful opioid and other substance use, (2) perceptions of others’ substance use, (3) lived experiences of opioid and other substance use, (4) news reports or papers related to opioid and other substance use, (5) health care initiatives, (6) adverse effects on children’s health due to parental substance use, and (7) topics related to nonopioid substance use. Among these 7 themes, our user engagement analysis revealed that themes 4 and 5 received the highest average retweet counts, and theme 3 received the highest average tweet reply count. We further found that different computational methods excel in analyzing different themes.

**Conclusions:**

Social media platforms such as X can serve as a valuable tool for analyzing real-time discourse and exploring public perceptions, opinions, and behaviors related to maternal substance use, particularly, harmful opioid use in the perinatal period. More health promotion strategies can be carried out on social media platforms to provide educational support for the OUD perinatal population.

## Introduction

In 2021, it was reported that fatal drug overdoses accounted for 106,699 deaths in the United States, which marks a 14% increase in fatal drug overdose rates compared to the rates in the previous year [1]. Notably, harmful opioid use was a major contributor, implicated in 75.36% of these drug overdose fatalities [1,2]. The ongoing opioid epidemic persists as a significant public health challenge in the United States, particularly affecting individuals of reproductive age. It is estimated that 70% to 80% of women with substance use disorder (SUD) have concurrently endured physical abuse, sexual harassment, and severe mental health conditions—factors that exacerbate morbidity and mortality rates in this demographic [3]. Moreover, in 2019, about 20% of birthing individuals reported their opioid misuse during pregnancy based on their self-reported data [4]. According to self-reported data from 2019, disseminated by the Centers for Disease Control and Prevention [4], approximately 7% of surveyed individuals indicated they used prescription opioid pain relievers while pregnant, with one-fifth of them reporting opioid misuse. These findings underscore the prevalence and detrimental impact of opioid use disorders (OUDs) among pregnant and postpartum individuals, making it one of the most prevalent and pernicious forms of SUD in the United States.

In the context of perinatal care, opioid medications are often prescribed for pain relief (analgesia). Nonetheless, the prolonged use of these prescription opioids can cause dependency, tolerance, and addiction, collectively characterized under OUD. Data from 2017 to 2018 indicate that approximately 42.5% of opioid overdose fatalities among women in the United States were associated with prescription opioids [3]. Given their efficacy in pain management, a significant proportion of women diagnosed with OUD are more inclined to persist in their opioid consumption throughout pregnancy. The excessive prescription of opioids during this critical period not only heightens the susceptibility to OUD in new mothers but also escalates the likelihood of postpartum depression [5]. Furthermore, the deleterious impacts of opioid use extend to fetuses, notably increasing the risk of neonatal opioid withdrawal syndrome [6], seizures, and respiratory complications in neonates [5]. Despite the progress in medication-assisted treatments designed to enhance maternal and fetal health outcomes by mitigating the fetal stress associated with OUD, the ramifications of opioid use during prenatal and postpartum phases persist as a significant societal concern [5].

The ubiquity of social media plays a key role in facilitating public discourse and fostering connections, thereby making these platforms invaluable for real-time public data collection and capturing public sentiment, increasingly recognized as vital for public surveillance. X, formerly known as Twitter, emerged as a preeminent social media platform for its expansive global reach, enriched by its user engagement features and capabilities for users to rapidly disseminate information through retweets. By 2023, X had attracted more than 450 million active users monthly [7], highlighting its significance in global digital communication. In addition, research also reveals that X is among the foremost platforms used by individuals for stress management through constructive and detrimental practices, as well as expressing views, concerns, and insights about diverse health issues [8]. Furthermore, X represents a cost-effective and efficient medium for the dissemination of public health communications, providing a rich vein for researchers to identify consumer health interests and concerns [8-10]. Its user engagement functions, such as retweets, likes, and user follow-up conversations, enable the dynamic assessment of public sentiment, facilitate the real-time exchange of personal health perspectives, and enhance public education through the accessible presentation of scientific findings [9,11,12].

The exploration of linguistic patterns within X conversations related to opioids unravels intricate insights into harmful opioid use trends and societal perceptions of this substance. Graves et al [13] analyzed 84,023 opioid-related tweets in the United States, delineating discussions on opioid issues across diverse geographic regions. Their analysis indicated that ongoing monitoring of X data could facilitate the detection of emerging opioid trends. Moreover, this study highlighted that linguistic analyses may yield valuable insights into public reactions to dynamic opioid markets and identify intervention opportunities for targeted high-risk populations and areas [13].

Existing literature, primarily using qualitative methodologies and randomized controlled trials with limited samples, has predominantly focused on risk factors associated with opioid use during pregnancy, perceptions of treatment for OUD, and health care–seeking experiences of women with OUDs [14-16]. Scarce studies used X data to explore widespread perception related to OUD, its treatments, and naloxone [16]. To the best of our knowledge, there is a void in social media research examining perinatal OUD to gauge public sentiment. As such, in this study, we address this gap by analyzing tweet content on opioid use in the perinatal period during the opioid epidemic from 2019 to 2021. Through classical thematic analysis, a methodology extensively used in qualitative research for identifying, analyzing, and reporting data patterns [17] along with the latest machine learning models for initial testing the feasibility of scaling up the annotations and improving labeling accuracy, we aim to uncover emerging perinatal OUD trends and user engagement reflected in social media discourse [18-20]. More specifically, the objective of this study was to unravel X users’ disclosure and communication patterns concerning public opinions, personal experiences, and challenges associated with OUD and OUD care during pregnancy and postpartum periods, with a view of enhancing health promotion strategies for this vulnerable population on social media platforms. Consequently, this study provides a more detailed overview and insightful implications on public health policy and services, aiming to enhance OUD treatment options for women in the perinatal period. As such, our three primary research questions (RQs) to guide this study are as follows:

- RQ1: What are key themes and trends in perinatal OUD discussions on X from 2019 to 2021?
- RQ2: What user engagement patterns, including disclosure frequency, retweeting and replying tweet behaviors, emerged in perinatal OUD–related discourse on X?
- RQ3: Considering the labor-intensive process of manually coding qualitative data, even for a small sample of tweets, are there any computational methods to streamline and scale this annotation effort?

## Methods

### Ethical Considerations

The University of South Carolina’s Institutional Review Board (Pro00113240) has reviewed this study’s data collection and analysis approach. Since we extracted public data from X, this study was deemed as nonhuman participant research and exempted from review. Furthermore, we also cleaned and de-identified anonymous tweets before our data analysis process.

### Data Collection

**Figure 1:**
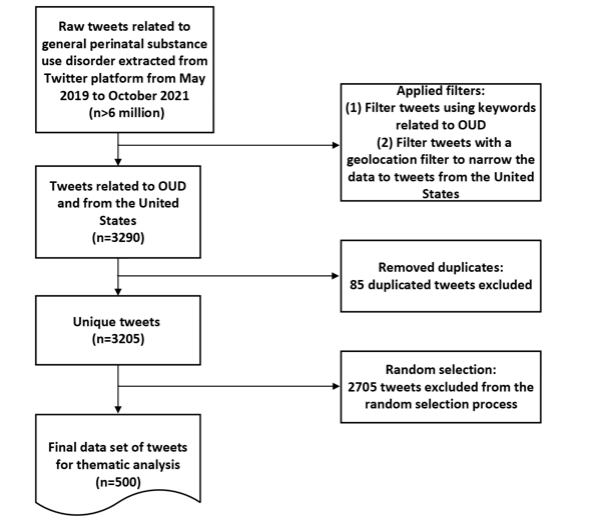
Flowchart of data collection, cleaning, and sampling process. OUD: opioid use disorder.

We gathered 6 million raw tweets from X related to general perinatal OUD guided by a set of keywords provided by our clinical team, spanning from May 2019 to October 2021. This data set underwent a refinement process using English keywords pertinent to OUD in English, encompassing terms such as “opioid,” “heroin,” “codeine,” “oxycodone,” “Vicodin,” “fentanyl,” and “Percocet,” among others. The full list of these keywords is listed in Multimedia Appendix 1. Additionally, we applied a geolocation filter to narrow down the data to tweets within the United States. This filtering process yielded a narrowed pool of 3290 perinatal opioid use–related tweets. Then, we further identified and removed 85 duplicate tweets to 3205 unique tweets. A random sample of 500 was then extracted for thematic analysis, a sample size aligned with prior qualitative research methods that have analyzed Twitter data sets of similar scope [21,22]. Figure 1 outlines the sequential steps of data collection, cleaning, and sampling processes used in this study.

**Figure 2:**
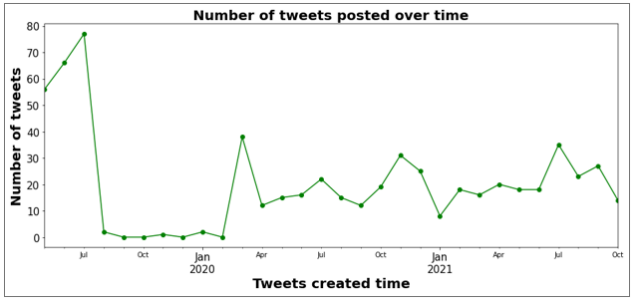
Number of monthly perinatal opioid use disorder–related tweets from May 2019 to October 2021.

Figure 2 illustrates the trends of the number of monthly perinatal OUD-related tweets from May 2019 to October 2021. The highest volume of tweets was recorded in July 2019. Subsequently, from August 2019 to February 2020, there was a significant decline in the number of tweets. In March 2020, coinciding with the COVID-19 period, the tweet volume surged nearly 7-fold.

### Thematic Analysis

Two trained researchers independently conducted the thematic analysis on the 500 randomly selected tweets, achieving a satisfying κ score of 0.82. NVivo (version 12; Lumivero) software was used in analyzing the tweet data set through a manual coding process, such as labeling and categorizing specific sections [23]. Using open-coding techniques, the researchers identified themes in the X discourse on opioid use and labeled codes based on the content and context of the tweet. Each tweet was coded independently, and the main themes identified were further divided into subthemes. These subthemes provided specificity and helped address the tweet’s context and information. The occurrence of thematic overlap was notable, necessitating multiple categorizations per tweet to encapsulate the breadth and complexity of themes articulated [24]. In total, the analysis of the 500 tweets resulted in the identification of 7 main themes. The researchers deployed 174 unique codes throughout this process, accounting for 1307 references across the selected tweets.

## Results

### Identified Themes of Perinatal OUD

Table 1 provides a brief description of the 7 main themes, the number of references for each theme, and their sample tweets. The number of references in NVivo refers to the number of times a passage of text was coded to each theme and subtheme. The seven primary themes we identified include (1) political views related to harmful opioid and other substance use, (2) perceptions of others’ substance use, (3) lived experiences of opioid and other substance use, (4) news reports or papers related to opioid and other substance use, (5) health care initiatives, (6) adverse effects on children’s health due to parental substance use, and (7) topics related to nonopioid substance use. We described each main theme and its subthemes in the following subsections.

**Table 1:** Seven main themes identified with opioid use disorder in the perinatal period.

Main themes	Description	Sample tweet	References (n=457), n (%)
Political views related to harmful opioid and other substance use	Opinions or perceptions on the influence of politics and other governmental structures on substance use	“@***a@real*** @*** @*** was born in Detroit! ***, you need to go back to Pennsylvania and, those suburban Opioid addicts in your old neighborhood.”	101 (22.1)
Perceptions of others’ substance use	Opinions or perceptions on other people’s use of substances	“@***a @***a @***a @***a @***a Can we talk seriously about opioid addiction in pregnant mothers? How We the PEople can write law to PROactively PROtect our women & children? @***a @***a @***a Get to the core”	91 (19.9)
Lived experiences of opioid and other substance use	Personal substance user’s experiences including individual’s experience or an individual’s family or friends	“I Am: An Un-apologetic Opioid Patient – National Pain Report ...” “Chronic pain is a disease. Do we have to start a mea culpa for every disease? Cancer, MS, polio, broken bones, DDD, dystonia, strokes, Parkinsons, IBS, kidney stones, childbirth w/o a spina”	70 (15.3)
News reports or papers related to opioid and other substance use	Published information regarding a recent event, change, or situation related to substance use	“Rate of women addicted to opioids during pregnancy quadrupled in 15 years, CDC says”	48 (10.5)
Health care initiatives	Information and methods for either educational purposes or to address current health needs	“Opioid exposure following vaginal delivery appears to be a trigger for future persistent opioid use and misuse, independent of confounding factors #***a #SOAPAM2019 #BestPaper #OBAnes #OpioidCrisis #OpioidEpidemic”	37 (8.1)
Adverse effects on children’s health due to parental substance use	The consequences that children face due to their parents ’ use of substances	“Not to be stupid but does that go for heroin crack marijuana ok to put as much in your body as you want. My wife had an abortion we regret it now you have to live with it”	24 (5.3)
Topics related to nonopioid substance use	Tweets comparing other substances or ideas to opioids or about nonopioid drugs	“Booking holidays and city breaks - that’s my heroin. Edinburgh, Brussels, Manchester & Berlin done! Roll on Vegas, Portugal & Spain! See the world, have great fun with great friends and family! Get her booked! #***”	86 (18.8)

^a^***: Reaction from a private Twitter user account.

### Political Views Related to Harmful Opioid and Other Substance Use

Tweets were categorized under the theme “political views or criminalization of behaviors related to opioid and other substance use” if the content referred to an individual’s beliefs or opinions on a political or governmental topic related to opioids or other substances. Within this theme, the researchers identified 2 major subthemes, as shown in Table 2, with “opioid epidemic” being the most common (n=68). Under the “opioid epidemic” subtheme, tweets frequently referenced opioid addiction (n=11), followed by illegal drug importation (n=10), and companies that manufacture opioids (n=8). Meanwhile, tweets categorized under the “multifactorial perspectives” subtheme mentioned the topic of abortion most often (n=7), followed by drug addiction (n=4) and human trafficking (n=4).

**Table 2:** Subthemes of political views related to opioid and other substance use.

Subthemes	Description	Sample tweet	References (n=89), n (%)
Opioid epidemic	Political opinions regarding the opioid crisis	“Why don’t we look at maternal health disparities the way we do the opioid epidemic? We don’t blame that community for addiction, we look at other factors and causes (prescribing, etc). What are risk factors in the community leading to maternal disparities?”	68 (76)
Multifactorial perspectives (abortion, child abuse, drug addiction, homelessness, human trafficking, and imprisonment)	Tweets that contain multiple components within one tweet in relation to politics	“@***^a^ @*** NYC is becoming a #[obscenity] $15 coffee, gangs running some neighborhoods, illegals [obscenity] taxpayers money dry, reduction in services for residents, homelessness, drug addiction, human trafficking, child abuse #ToxicLiberals advocating 4 everything but values integrity and Americans”	21 (24)

^a^***: Reaction from a private Twitter user account.

The 21 tweets categorized under the “multifactorial perspectives” subtheme were further categorized into 4 sublevels: individual, general public, health care provider, and policy. The result is presented in Table 3. The analysis revealed policy-level tweets as the most frequently occurring with a count of 14, followed by general public-level tweets (n=7). However, no tweets were at the health care provider (n=0) or individual (n=0) levels. Policy-level tweets were defined as tweets referring to specific governmental regulations or policies, with most tweets about abortion and domestic terrorism. General public tweets were defined as tweets on the general public, with no particular attention on governmental policies. Within this category, most tweets were about drug addiction and homelessness. Coding for the general public tweets that target no specific audience is important, as it facilitates researchers in comprehending the perspectives and beliefs on current issues, thereby suggesting potential solutions.

**Table 3:** Sublevels of multifactorial perspectives.

Levels	Description	Sample tweet	References (n=21), n (%)
Policy level	Tweets referring to specific governmental regulations or policies	“i can’t believe this people haven’t been prosecuted for domestic terrorism yet. what a [obscenity] corrupted [obscenity] system. a child molester walking amongst the children and a [obscenity] heroin addict/porn star acting as a journalist. [obscenity] white people”	14 (67)
General public level	Tweets on the general public, with no particular attention on governmental policies	“this is their concern when homeless line streets of major cities, when illegals flood our country, when infrastructure needs repair, when babies left to die after birth if unwanted, when illegal voting plagues the system, when hundreds die from opioid addiction, etc. really?!”	7 (33)

### Perceptions of Others’ Substance Use

Tweets were classified under the “perceptions of others’ substance use” theme when their content demonstrated an individual’s opinions regarding another person’s substance use or general use in the larger population. The researchers identified 5 major subthemes. The most common subtheme was “opioid use” (n=34), followed by “children exposure” (n=21), “substance use during pregnancy” (n=20), “polysubstance use” (n=8), and “religious beliefs” (n=6), as shown in Table 4.

**Table 4:** Subthemes of perceptions of others’ substance use.

Subthemes	Description	Sample tweet	References (n=89), n (%)
Opioid use	The use of opioids, prescribed or illicit use	“@***a is surprised by the prevalence of Opioid prescribing after a vaginal delivery: 30%; 87% after C Section. Most patients rate pain at 0 at D/C and do not use all the opioids they are prescribed.”	34 (38)
Children exposure	Children being exposed to substances, intentional or unintentional	“First, how dare you even think of giving a baby Morphine?!! I’m beyond [obscenity] 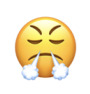 .”	21 (24)
Substance use during pregnancy	The use of substances during which a fetus is developing in the mother’s womb	“@***’d rather have 95% of your profits...” “Momma’s got baby number seven on the way and has an opioid craving she needs to take care of ...”	20 (22)
Polysubstance use	The use of more than one substance simultaneously	“Forgot to mention: his default state is ‘vapes maximum strength THC 24/7’ recovering opioid addict still on prescription opioids, also ‘occassionally’ does coke” “His drug problem is clearly SKY HIGH atm so yeah, baby intervention tonight”	8 (9)
Religious beliefs	Tweets regarding an individual’s religious views or beliefs	“PRAYER REQUEST: 100 percent disabled vet in his last moments from mesothelioma (service related-asbestos exposure.) He is a warrior born/bred.” “He’s in great pain; being kept on morphine to keep him comfortable. He can’t speak, eat or move.” “Pray for a peaceful transition. Amen”	6 (7)

^a^***: Reaction from a private Twitter user account.

### Lived Experiences of Opioid and Other Substance Use

Tweets that described an individual’s experience or experience of family and friends related to opioid and other substance use fell under the main theme of “lived experiences of opioid and other substance use.” There were 2 main subthemes, “opioid use” (n=65) and “polysubstance use” (n=5), identified under this category, as shown in Table 5.

**Table 5:** Subthemes of lived experiences of opioid and other substance use.

Subthemes	Description	Sample tweet	References (n=67), n (%)
Opioid use	The use of opioids, prescribed or illicit use	“It’s been a year since I lost my baby brother to dumb ass heroin and my heart still hurts just the same. I miss you so much”	62 (92)
Polysubstance use	The use of more than one substance simultaneously	“What’chu want? I got ketamine, MDMA, Adderall, Bromo-Dragonfly, heroin, coke, crack, codeine, oxys, percs, vikes, PCP, LSD, Dilaudid, mescaline, mushrooms, bath salts, cortisone, Toradol. I got molly. I got her sister Sandra. I got big Frank. I got birth control, I got Plan B.”	5 (8)

Most of the tweets under “opioid use” were related to the loss of loved ones, predominantly due to harmful opioid use leading to fatal overdose (n=24), followed by opioid use for pain management (n=17). When further stratified by opioid type mentioned under both subthemes combined, heroin was the most common (n=17), followed by morphine (n=10), codeine (n=10), fentanyl (n=9), oxycodone (n=5), and hydrocodone (n=2).

### News Reports or Papers Related to Opioid and Other Substance Use

Tweets that referred to published news reports or papers concerning opioid and other substance use were placed under “news reports or papers related to opioid and other substance use” theme. Five subthemes were identified, as shown in Table 6, with “opioid use” being the most common (n=30), followed by “substance use during pregnancy” (n=11), “unintentional drug exposure” (n=2), “child neglect” (n=2), and “concurrent use of alcohol and opioids” (n=2).

**Table 6:** Subthemes of news reports or papers related to opioid and other substance use.

Subthemes	Description	Sample tweet	References (n=47), n (%)
Opioid use	The use of opioids, prescribed or illicit use	“***^a^ hospital shut down as revelations emerge that a dangerous dose of morphine killed 7-month-old baby Ethan. We tell you more on #K24EveningEdition”	30 (64)
Substance use during pregnancy	Prenatal substance use during fetus development	“The opioid crisis comes to the classroom as soaring numbers of children born in drug withdrawal reach school age | The Seattle Times”	11 (23)
Unintentional drug exposure	Accidental exposure of children to substances	“highlights problems: Baby was exposed to 87 micrograms, 4% of the therapeutic dose. Codeine level tho was ‘stratospherically high’: 50x higher than would be expected. Opioid poisoning could not be the cause of death. Neonatal opiod toxicity hard to envision.”	2 (4)
Child neglect	A form of abuse in which the caregiver fails to meet the child’s basic need	“Police arrested Edwardo Zepeda after he lost his daughter when he went on a late-night alcohol run. The 4-year-old was later found sleeping in between two power boxes. He is now charged with child abuse @KTNV”	2 (4)
Concurrent use of alcohol and opioids	The use of opioids and alcohol simultaneously	“Radle was born in Tulsa, Oklahoma, and died in May 1980 from a kidney infection, exacerbated by the effects of alcohol and narcotics; he was 37.”	2 (4)

^a^***: Reaction from a private Twitter user account.

Tweets were also stratified by the news media outlets in which they were published and categorized by national outlet, state outlet, community, health news, international, and unknown source. News reports or papers generated at the national level included CNN, USA Today, New York Times, Fox News, and ABC News, which provide information and reports on the United States overall. State-level news outlets included Oklahoma News, Seattle Times, Tennessean, and San Antonio Express-News. Community-level news outlets, such as Vox News, are privately owned, publish opinionated journals and cover American news. Tweets listed under health news are news outlets that specifically publish medically related information and reports, such as *Science Daily*, *Medical Press*, and *JAMA Network*. International-level news is international news outlets, which include Irish News, Irish Times, and United Press International. Finally, tweets were coded as “unknown” if the user-generated content referred to a news report or paper but did not specify its source. Among these 6 categories, national-level news and unknown were the most common (n=13), followed by state-level news (n=9), international-level (n=5), health news (n=5), and community-level news (n=2).

### Health Care Initiatives

Tweets were categorized under “health care initiatives” theme if they contained information to educate the public and address current societal health needs. Four main subthemes were identified under this category as shown in Table 7. The most common subtheme was “speaker presentations” (n=20), followed by “government initiatives” (n=6), “research” (n=6), and “addiction treatment services” (n=3).

**Table 7:** Subthemes of health care initiatives.

Subthemes	Description	Sample tweet	References (n=35), n (%)
Speaker presentations	Individuals or a group of individuals who educate others and raise awareness based on current data and information	“Important #***^a^ findings by @*** on the risk of persistent opioid use after childbirth, especially after c-sections and when getting prescriptions before delivery.”	20 (57)
Government initiatives	Government-funded programs or policies to assist with substance use	“During Health, Welfare Family Services cmte, Senate Committee Chairman @***Senate discusses @*** HEAL opioid research grant working in 16 counties, two of them in his district.”	6 (17)
Research	Ongoing investigation or discoveries regarding substance use	“PROMISE and MOTHER studies debunked lots of myths—dose of #buprenorphine and #methadone not related to neonatal withdrawal severity and methadone was associated with more #opioid need than bup Hendree Jones #CPDD19”	6 (17)
Addiction treatment services	Treatment programs with the objective to assist people with drug addiction	“Great tour and presentation from the staff at *** Addiction and Family Services. We were impressed with the organizations no wait time screening and triage as well as the Rapid Access Opioid Treatment program. Providing services to those when they need it most is crucial”	3 (9)

^a^***: Reaction from a private Twitter user account.

Tweets placed under “speaker presentations” were further divided into national, state, and clinician levels. National presentations were cited most frequently (n=10), followed by clinician speaker presentations (n=5), and finally, state-level presentations (n=3).

All sample tweets coded under “health care initiatives” were also stratified in relation to opioid use during prenatal care, pregnancy period or delivery, postpartum or breastfeeding, and childcare or maternal health to determine the prevalence of perceptions and opinions about opioid use across the different stages of pregnancy. The results showed tweets were most commonly about childcare and maternal health (n=11), followed by the quality of care received during pregnancy and delivery period (n=7), care quality postpartum and during the breastfeeding period (n=4), and finally, prenatal period (n=1).

### Adverse Effects on Children’s Health due to Parental Substance Use

Tweets that described the outcomes of children’s health due to parental use of opioids and other substances were listed under “adverse effects on children’s health due to parental substance use” theme. Table 8 indicates the most common subtheme under this category was “multifactorial perspectives” (n=22), followed by opioid orphans (n=1).

**Table 8:** Subthemes of adverse effects on children’s health due to parental substance use.

Subthemes	Description	Sample tweet	References (n=23), n (%)
Multifactorial perspectives (neonatal abstinence syndrome, abortion, miscarriage, foster care, and deaf)	Tweets that contain multiple components within one tweet in relation to adverse effects on children	“Was born in withdrawal to heroin. Had 2 ab, tubes tied after 3rd child. Have company spons ins, still can’t afford to go to dr even though have afib, so went strict vegan, quit weed, liquor, etc. Have been on welfare b4, but now help family, but I’m happy and grateful!”	22 (96)
Opioid orphans	Children who became parentless due to parental opioid use	“15-year-old ***^a^ calls himself an opioid orphan, which is a child who has lost one or both parents to the opioid crisis whether it’s to rehab prison or death. Part of Steven’s path to healing is his plan to help other kids.”	1 (4)

^a^***: Reaction from a private Twitter user account.

Under “multifactorial perspectives,” tweets related to neonatal abstinence syndrome were the most frequent (n=9), followed by abortion (n=5), foster care (n=2), miscarriage (n=1), and deaf (n=1). There was 1 specific tweet about opioid orphans, which is a term describing children who become parentless due to parent’s use of opioid (n=1).

### Topics Related to Nonopioid Substance Use

Tweets were coded as “topics related to nonopioid substance use” if the content was not related to opioid use. There were references to other drugs, abstract ideas about opioids, and mentions of opioids in pop culture, music, and television. There were 3 major subthemes under this category, with the most common being nonopioid drugs (n=43), followed by pop culture (n=28), and Norco, California (n=6), as listed in Table 9.

**Table 9:** Subthemes of topics related to nonopioid substance use.

Subthemes	Description	Sample tweet	References (n=77), n (%)
Pop culture	Entertainment that is enjoyed by the general public	“Book your tickets to see our Grad Show ‘Darknet’ 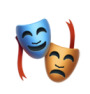 . 10/13July at The Union Theater.” “Computer hacking, Heroin overdosing, Methadone Selling, The rise of Robots and Artificial Intelligence, Family conflict and Big tech ... ”	28 (36)
Nonopioid drugs	In relation to drugs that are not opioids	“A few people have asked about the #surgery I had.” “It was a #laparoscopic #hysterectomy with a #chemo flush. I have three incisions in my #stomach and they took my #cervix #FallopianTubes and #uterus out through my #vagina. #no #narcotics #cannabis only!”	43 (56)
Norco, California	In relation to the city in California with the same name as the brand name of hydrocodone or acetaminophen	“***a going away party. It [obscenity] that one of my best friends is moving so far away. But I wish nothing but the best for him and his family. I owe a lot to him. A real to friend! @ Norco, California”	6 (8)

^a^***: Reaction from a private Twitter user account.

Table 10 presents the distribution of various identified topics or themes across 3 time periods: before COVID-19 in 2019, during the COVID-19 pandemic period without COVID-19 vaccination (year 2020), and during the COVID-19 pandemic period with COVID-19 vaccination (year 2021). Notably, in the pre–COVID-19 era, the predominant theme among tweets pertained to “political views related to substance use.” Throughout the duration of the COVID-19 pandemic, “lived experiences of opioid and other substance use” emerged as the theme with the highest frequency of tweets, closely followed by “political views related to substance use.” In the COVID-19 pandemic period after launching the COVID-19 vaccination in 2021, “lived experiences of opioid and other substance use” continued to dominate the thematic landscape of the tweets.

**Table 10:** Comparison of various theme distributions during 3 time periods of before and during the COVID-19 pandemic with and without COVID-19 vaccination.

Themes	Time period (n=600)		
	Pre–COVID-19 tweets, n (%)	COVID-19 without vaccination tweets, n (%)	COVID-19 with vaccination tweets, n (%)
Political views related to opioid and other substance use	63 (10.5)	54 (9)	46 (7.8)
Perceptions of others’ use	28 (4.7)	26 (4.3)	22 (3.7)
Lived experiences of opioid and other substance use	30 (5)	71 (11.8)	72 (12)
News reports or papers related to opioid and other substance use	19 (3.2)	14 (2.3)	24 (4)
Health care initiatives	13 (2.2)	13 (2.2)	10 (1.6)
Adverse effects on children’s health due to parental	17 (2.8)	14 (2.3)	14 (2.3)
Topics related to nonopioid substance use	30 (5)	8 (1.3)	12 (2)

To understand user engagement dynamics on X, we further analyzed additional users’ retweet and reply tweets to examine user interaction behaviors. Table 11 summarizes the mean counts and percentages of retweets and replies associated with tweets across the identified thematic categories. Analysis of user engagement in terms of retweeting behavior reveals that “health care initiatives” emerges as the preeminent theme, recording the highest mean retweet count, closely followed by “news reports or papers related to opioid and other substance use.” In terms of tweet replies, “lived experiences of opioid and other substance use” was ranked the highest replied theme, with other thematic categories lagging significantly.

**Table 11:** Average retweet and reply count based on themes.

Themes	Theme retweet count, mean (SD)	Tweet reply count, mean (SD)
Adverse effects on children’s health due to parental substance use	0.98 (3.26)	0.51 (0.99)
Health care initiatives	1.61 (2.28)	0.31 (0.47)
Lived experiences of opioid and other substance use	0.47 (1.83)	1.72 (5.05)
News reports or papers related to opioid and other substance use	1.47 (4.68)	0.40 (0.96)
Topics related to nonopioid substance use	0.32 (0.77)	0.34 (0.89)
Perceptions of others’ substance use	0.26 (0.79)	0.53 (0.92)
Political views related to harmful opioid and other substance use	0.62 (2.81)	0.46 (1.16)

Table 12 presents the distribution of tweets across various themes as percentages. Importantly, the themes “lived experiences of opioid and other substance use” and “political views related to harmful opioid and other substance use” were ranked a higher percentage of the perinatal OUD tweet data set compared to the others.

**Table 12:** Thematic breakdowns of perinatal opioid use disorder tweets in percentages.

Themes	Tweet count (n=600), n (%)
Political views related to harmful opioid and other substance use	163 (27.2)
Perceptions of others’ substance use	76 (12.7)
Lived experiences of opioid and other substance use	173 (28.8)
News reports or papers related to opioid and other substance use	57 (9.5)
Health care initiatives	36 (6)
Adverse effects on children’s health due to parental use of substances	45 (7.5)
Topics related to nonopioid substance use	50 (8.3)

## Discussion

### Principal Findings

Overall, public health researchers and public health authorities can potentially use these social media platforms as a communication and monitoring tool to promote public health awareness and positively influence public health behavioral changes in all pregnancy stages [25,26]. This study leveraged the expansive reach of X with a set of tweets pertinent to perinatal SUD as informed by relevant keywords curated by our clinical team, ranging from May 2019 to October 2021. This corpus underwent a rigorous refinement procedure to result in a set of 3205 unique perinatal OUD-related tweets in the United States, among which we randomly selected 500 tweets for in-depth thematic analysis, forming the foundation for our data analysis.

Our thematic analysis unveiled several key trends: the consequences of harmful opioid use in the perinatal period, shared experiences and published newspapers on opioid use, perspectives on the opioid epidemic, and current protocols and practices that are put in place to combat the opioid epidemic. As one of the first such studies to explore perinatal OUD disclosure patterns on social media, we identified seven major themes from 500 random tweets related to OUD in the perinatal period: (1) political views related to harmful opioid and other substance use, (2) perceptions of others’ substance use, (3) lived experiences of opioid and other substance use, (4) news reports or papers related to opioid and other substance use, (5) health care initiatives, (6) the adverse effects on children’s health due to parental substance use, and (7) topics related to nonopioid substance use.

This study also revealed the potential of social media platforms such as X for public health surveillance. For example, before the COVID-19 pandemic, the dominant theme about OUD in the perinatal period was “political views on substance use.” However, during the prevaccination period in the pandemic, tweets about “lived experiences of opioid and other substance use” and “political views related harmful opioid and other to substance use” surged among tweets related to OUD in the perinatal period, and they remained prominent even after COVID-19 vaccinations became available. This study further provides support that social media data can provide valuable insights and trends regarding public concerns and opinions during public health crises. Policy makers can leverage these insights and trends emerged from social media platforms to understand evolving public concerns and opinions and further develop public health policies and interventions accordingly.

Overall, these themes collectively identified socioecological factors at the individual, interpersonal, environmental, community, and national levels (including policies and structures that can promote or hinder treatment), underscoring the multifaceted nature of OUDs and their treatment within the perinatal population. Addressing 3 issues necessitates a multitiered treatment strategy that fosters substantial and sustainable changes, advocating a systematic approach for collective action among key stakeholders. Notably, the analysis highlighted the prominence of “health initiatives” conducted by health welfare, family services, senate committees, local addiction treatment services, and other legislative bodies. The health promotion strategies funded by government health programs can be used to increase public awareness on social platforms about adverse effects caused by OUD and disseminate information on safe nonpunitive networks where pregnant and postpartum women can safely access treatment. There are also opportunities to disseminate targeted research discoveries, especially to and for families and children. This study underscores the potential of government-initiated health promotion strategies through social media platforms, such as X, particularly for families and children impacted by OUD to enhance the quality of OUD care during the perinatal period.

Given the labor-intensive nature of the human manual annotation process, to elevate the scalability and precision of thematic analysis within perinatal opioid–related tweets, we also initially explored support vector machine (SVM), random forest (RF), and extreme gradient boosting (XGBoost) algorithms in tandem with the Word2Vec model, creating binary weak labeling models for each identified theme. These models were trained on 80% (n=400) of the manually annotated tweet data, using the derived themes as labels, and their performance was evaluated on the remaining 20% (n=100). The comparative analysis across 7 thematic categories revealed distinct strengths among the models: XGBoost demonstrated superior accuracy in themes concerning adverse effects on children’s health due to parental substance use, SVM excelled in analyzing health care initiatives and lived experiences of opioid and other substance use, and RF was significantly effective in interpreting politically charged content. This machine learning approach is promising to facilitate the processing of a larger data set but also enabling nuanced analysis across various dimensions, underscoring the potential of enhancing text analysis efficiency.

### Limitations

This research study had several limitations and challenges. First, key term ambiguity sometimes led to the inclusion of irrelevant data, such as tweets referring to Norco, California, coincidentally captured as the brand name of hydrocodone or acetaminophen. We had to manually exclude these irrelevant terms for our content analysis. Second, the anonymous nature of X posed challenges in consistently delineating the demographic and geographical details of users. Finally, the user-generated content provided limited insights into ethnic and racial disparities and maternal disparities in our small sample, which could be expanded with a much larger tweet sample in future studies.

### Discussions and Future Research

Addressing harmful opioid use in the perinatal period remains a public health imperative in the United States. Researchers have turned toward using X, a popular social media platform, to dissect health perceptions and behaviors, offering a lens on the opioid epidemic’s evolving discourse. Our study findings suggest a universal opioid screening in perinatal care, destigmatization, enhanced care access, and a preference for treatment over penalization to combat the epidemic effectively, especially among pregnant individuals. Using X as a valuable social media platform, we were able to analyze real-time public perceptions and behavior as well as current trends on the opioid epidemic and opioid use during the perinatal period. As such, this study provides important implications on perinatal OUD treatment policies, services, and potential ways of using social media as a platform to facilitate and promote healthy and impactful initiatives and community support to the vulnerable perinatal OUD population at large.

With many more identified relevant and touching themes, we significantly enriched the overall understanding of the complexity of OUD use in the perinatal care domain and associated pressing issues in a much broader way, calling for more research and services in this specific domain. First, this study highlights the need to establish more comprehensive initiatives and universal screening, as they were recommended by the American College of Obstetricians and Gynecologists and Centers for Disease Control and Prevention. Universal screening and assessment of all pregnant individuals for substance use are crucial during pregnancy, and targeted support for child welfare and maternal health, especially given that there are fewer initiatives on postpartum or breastfeeding and prenatal period, and thus there is room for expanding this area of care services, health promotion initiatives, and research. Second, we advocate for a collaborative and resource-adequate approach to mitigate OUD’s impacts on maternal and child health [4,27,28]. Likely, social media platforms can be used as a medium to promote such universal screening, reduce stigma, and increase assisted treatment programs for these disadvantaged vulnerable populations through government agencies, public health–related organizations, and communities. Third, more government initiatives and services to address the health concerns of pregnant individuals with OUD delay in health seeking, an emphasis might need to be placed on treatment rather than criminalization of child abuse and loss of child custody [10]. Fourth, additional studies assessing racial and ethnic maternal disparities and maternal disparities are needed due to significant racial and ethnic disparities with reported higher maternal morbidity and mortality rates for Black women compared to non-White Hispanic women due to pregnancy-related causes [29] and postpartum depression driven by increased substance use of alcohol and illicit drug use during pregnancy [30]. As a result, further research is essential to address racial and ethnic disparities, emphasizing more the important role of social media platforms in broadening outreach and support for vulnerable populations, guiding policy changes, and fostering stakeholder engagement for systematic solutions in OUD-related perinatal care.

Moreover, to improve the labeling accuracy and scalability, our future research will also aim to integrate large language models to refine the labeling technique, leveraging large language models’ advanced text comprehension for improved feature extraction and more accurate predictions, thereby streamlining the analysis of complex thematic data within social media discourse.

Overall, as topics related to perinatal OUD discussed on social media platforms evolve, more systematic investigation is needed to capture the full spectrum of perinatal opioid misuse and disparities. Larger samples in different geolocations from more diverse social media platforms may provide greater insights on current health perceptions and public response on opioid use in perinatal care for practical public health policy implications.

### Conclusions

Given the prevalence and detrimental impact of OUD among pregnant and postpartum individuals, this study aims to enhance our understanding of opioid use patterns during the perinatal period by analyzing related tweets on the X platform. We randomly sampled 500 tweets from May 2019 to October 2021 and conducted a thematic analysis.

Our analysis, thematic analysis, identified 7 key themes in perinatal OUD discussions, with “lived experiences of opioid and other substance use” and “political views related to harmful opioid and other substance use” among the most frequently discussed on X. Our user engagement analysis revealed that “health care initiatives” and “news reports or papers related to opioid and other substance use” received the highest average retweet count, and “lived experiences of opioid and other substance use” received the highest average tweet reply count. Given the labor-intensive nature of manual annotation, we investigated machine learning models to improve the scalability and precision of thematic analysis. We found that different computational methods excel in analyzing different themes: XGBoost for identifying adverse effects on children’s health due to parental substance use, SVM for analyzing health care initiatives and lived experiences of substance use, and RF for interpreting politically charged content.

## Appendix

Multimedia Appendix 1 Keyword list.

## Abbreviations

OUD: opioid use disorder
RF: random forest
RQ: research question
SUD: substance use disorder
SVM: support vector machine
XGBoost: extreme gradient boosting
