# Supplementary material for: Disclosure Patterns of Opioid Use Disorders in Perinatal Care During the Opioid Epidemic on X From 2019 to 2021: Thematic Analysis
Source: JMIR Pediatr Parent. 2024 Oct 7;7:e52735. doi: 10.2196/52735 (PMC11494255; doi:10.2196/52735)
Supplement: Multimedia Appendix 1 [file pediatrics_v7i1e52735_app1.docx]

### Multimedia Appendix 3

A full list of keywords related perinatal opioid use disorder.

**Primary Keywords**

Alcohol, Cocaine, Amphetamines, methamphetamine, Hallucinogens, nicotine, Opioid, sedatives, diazepam, Tobacco, SUD, Heroin, Cannabis, MDMA, LSD, weed, meth, XTC, benzodiazepines, stimulants, Morphine, Fentanyl, Codeine, drug, drug use.

**Secondary Keywords**

***- Perinatal Related***

childbearing, expecting, prenatal, Birth weight, pregnant woman, baby, infant, pregnancy, babyloss, baby loss, pregnancy loss, iam1in4, waveoflight, Pregnant, Preggers, Baby time, Childbirth, Giving birth, Newborn, Delivery, Abortion, Homebirth, Prenatal class, Miscarriage, Fetal, Stillbirth, Stillborn, Preterm, Postterm, Due Date, Baby loss, Neonatal, Neonatal Intensive Care, Neonatal Intensive Care Unit, Infant loss, Low birth weight, LBW.

***- COVID-19 Related:***

CORONA, corona, COVID-19, covid 19, covid, coronavirus, Coronavirus, Corona Virus, NCOV, sarscov2, sars cov2, c2019ncov, n95, ppeinfluenza

**- Disorder Related:**

Communication, Risky health behaviors, Inadequate nervous system, Memory issue, Poor behavior, outcome, rural, policy-relevant knowledge, Nutrition, feelings of anxiety, defect, abnormality, disorder, down syndrome, addiction, addict, racial disparity
